# Left egocentric neglect in early subacute right-stroke patients is related to damage of the superior longitudinal fasciculus

**DOI:** 10.1007/s11682-021-00493-w

**Published:** 2021-07-30

**Authors:** Barbara Spanò, Davide Nardo, Giovanni Giulietti, Alessandro Matano, Ilenia Salsano, Chiara Briani, Rita Vadalà, Claudia Marzi, Maria De Luca, Carlo Caltagirone, Valerio Santangelo

**Affiliations:** 1 Neuroimaging Laboratory, Santa Lucia Foundation (IRCCS Fondazione Santa Lucia), Via Ardeatina 306, 00179 Rome, Italy; 2 MRC Cognition and Brain Sciences Unit, University of Cambridge, Cambridge, UK; 3 Neuropsychology Center, Santa Lucia Foundation IRCCS, Rome, Italy; 4 PhD Program in Behavioral Neuroscience, Sapienza University of Rome, Rome, Italy; 5 NeuroRadiology, Santa Lucia Foundation (IRCCS Fondazione Santa Lucia), Rome, Italy; 6 Department of Clinical and Behavioral Neurology, Santa Lucia Foundation (IRCCS Fondazione Santa Lucia), Rome, Italy; 7 Department of Philosophy, Social Sciences & Education, University of Perugia, Perugia, Italy

**Keywords:** Neglect, Egocentric, Allocentric, Neuroimaging, VLSM

## Abstract

A typical consequence of stroke in the right hemisphere is unilateral spatial neglect. Distinct forms of neglect have been described, such as space-based (egocentric) and object-based (allocentric) neglect. However, the relationship between these two forms of neglect is still far from being understood, as well as their neural substrates. Here, we further explore this issue by using voxel lesion symptoms mapping (VLSM) analyses on a large sample of early subacute right-stroke patients assessed with the Apples Cancellation Test. This is a sensitive test that simultaneously measures both egocentric and allocentric neglect. Behaviourally, we found no correlation between egocentric and allocentric performance, indicating independent mechanisms supporting the two forms of neglect. This was confirmed by the VLSM analysis that pointed out a link between a damage in the superior longitudinal fasciculus and left egocentric neglect. By contrast, no association was found between brain damage and left allocentric neglect. These results indicate a higher probability to observe egocentric neglect as a consequence of white matter damages in the superior longitudinal fasciculus, while allocentric neglect appears more “globally” related to the whole lesion map. Overall, these findings on early subacute right-stroke patients highlight the role played by white matter integrity in sustaining attention-related operations within an egocentric frame of reference.

## Introduction

A common consequence of stroke in the right hemisphere is visuospatial neglect (‘neglect’), a complex syndrome associated with a reduced ability to orient towards the contralesional side of space. It is becoming increasingly clearer that neglect is not a monolithic disorder with a unique neural substrate, but rather a collection of symptoms (attentional bias towards the ipsilesional space, impaired spatial orienting in presence of competing stimuli, perceptual extinction, etc.) supported by different neural substrates (Vuilleumier, 2013). An important distinction has been drawn between space-based (egocentric) and object-based (allocentric) neglect (Robertson & Marshall, 1993). Egocentric neglect (EN) is characterized by failing to attend to the contralesional side of space with respect to a subject-centered view; conversely, allocentric neglect (AN) is characterized by failing to attend to the contralesional side of objects (Gainotti & Ciaraffa, 2013).

Whether EN and AN are related is still far from being understood. Behavioral studies demonstrated distinct patterns of deficit in EN and AN (Bickerton et al., 2011; Demeyere & Gillebert, 2019; Marsh & Hillis, 2008). While some patients neglect the contralesional hemispace only in terms of egocentric coordinates, other patients only in terms of allocentric coordinates (Demeyere & Gillebert, 2019). However, most patients show mixed forms of EN and AN, as evidenced by a failure to perceive both stimuli on the contralesional side of space, as well as on the contralesional side of objects (Demeyere & Gillebert, 2019). These findings make it difficult to understand whether EN and AN rely on independent or shared mechanisms. The existent literature based on neuroanatomical lesions does not substantially clarify this picture. Some findings suggest that EN/AN may be associated with distinct brain regions (Medina et al., 2009), while others indicate overlapping neural substrates (Rorden et al., 2012; Yue et al., 2012). A third possibility however, is that EN and AN might rely on both distinct and common patterns of gray and white matter lesions (Chechlacz et al., 2012).

The inconsistency amongst the above-reviewed findings might be due to the variety of tests used to classify the two symptoms (Saj et al., 2012). Neglect is routinely assessed with batteries of standardized tests (Azouvi et al., 2002; Halligan et al., 1991) that are useful to make a general diagnosis of neglect, but they do not typically allow to distinguish between EN and AN. To this purpose, a useful tool has proved to be the Apples Cancellation Test (ACT; Bickerton et al., 2011), which has been specifically set to detect general visual inattention and to differentiate EN/AN. The ACT can be considered as a sensitive task that allows to simultaneously measure within each patient both EN and AN.

To the best of our knowledge, only one study (Chechlacz et al., 2010) combined the assessment of EN/AN using the ACT with the investigation of their underlying neural substrates using a voxel-based lesion-symptom mapping (VLSM; Bates et al., 2003) analysis. Chechlacz and colleagues found that EN was associated with damage to middle frontal, postcentral, supramarginal, and superior temporal gyri. Conversely, AN was associated with damage to the posterior superior temporal sulcus, angular, middle temporal and middle occipital gyri. Damages to the intraparietal sulcus and temporo-parietal junction (TPJ), as well as white matter lesions, were instead associated to both forms of neglect (see also Chechlacz et al., 2012).

However, these relevant findings were based on a very limited number of cases, namely 11 patients with left EN and 8 patients with left AN. Furthermore, 6 of the patients included in that study showed both forms of neglect. However, in their VLSM analyses, Chechlacz et al. (2010) did not control for the potential influence of one form of neglect (e.g., allocentric) when assessing the other (e.g., egocentric), and vice versa. This leaves open the possibility that the neuroanatomical substrate associated with one form of neglect might have been confounded by the other. Finally, patients included in Chechlacz et al.’s study were in the chronic stage (i.e., > 9 months post-injury), while the relationship between EN/AN and lesions at earlier stages remains unexplored.

Here we leveraged on a larger sample of early subacute right-stroke patients (n = 100; assessed approximately 1-month post-injury) who underwent neuropsychological assessment for EN/AN (the ACT) and neuroimaging assessment of brain damage (VLSM). In agreement with previous literature showing that right-stroke patients with unilateral spatial neglect predominantly suffer from left side deficits (Vallar & Calzolari, 2018), we restricted our investigation to patients with left unilateral EN or AN. We conducted restrictive VLSM analyses that accounted for the variability due to allocentric performance while assessing egocentric performance, and vice versa. Moreover, we conducted a specific VLSM analysis on patients who showed both EN and AN. Overall, this approach allowed us to investigate the level of interdependency between the two forms of neglect, highlighting whether they rely on distinct or common damaged brain regions.

## Methods

### Participants

100 unilateral early subacute (according to Bernhardt et al., 2017; mean time of neuropsychological assessment since stroke onset = 23.0 ± 15.9; range 7–81 days) consecutive right-stroke patients hospitalized at the Santa Lucia Foundation were recruited for the study (Table 1). None of these patients suffered from general cognitive impairment (as assessed by a standard battery of several neuropsychological tests). All of them were administered the ACT. Twenty-four patients were excluded from the VLSM analysis: 7 because of neuroimaging scans of insufficient quality and 17 because they showed right EN and/or AN (i.e., negative score) at the ACT.

**Table 1:** Demographic information, Apples Cancellation Test scores, and neuroimaging assessment

	All patients recruited	Patients included in VLSM analysis
N	100	76
Age (years)	65.2 ± 14.2	64.6 ± 14.2
Sex (Male/Female)	52/48	36/40
Education (years)	10.2 ± 4.4	10.1 ± 4.4
Stroke to assessment interval days (mean ± SD) [range]	23.0 ± 15.9 [7—81]	23.8 ± 16.5 [7—81]
Apples Cancellation Test Egocentric scores (mean ± SD)	4.7 ± 6.3	5.8 ± 5.8
Apples Cancellation Test Allocentric score (mean ± SD)	5.1 ± 8.5	5.6 ± 8.2
CT/MRI (n)	65/35	52/24

### Apples Cancellation Test (ACT)

**Fig. 1:**
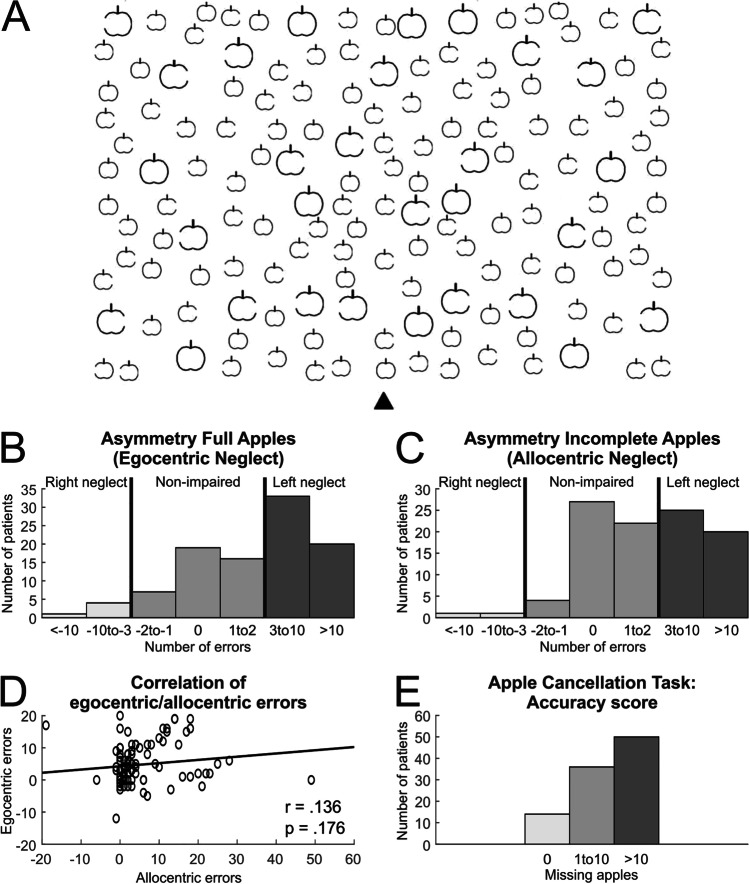
**A)** Copy of the page with apples used in the Apples Cancellation Test (Bickerton et al., 2011), where patients are asked to cross out all complete apples. **B)** Number of patients showing no impairment, left or right egocentric neglect, as evidenced by more missed targets (complete apples) on the contralesional side of the page vs. the ipsilesional side. **C)** Number of patients showing no impairment, left or right allocentric neglect, as evidenced by the cancellation of more left or right incomplete apples (i.e., distractors). **D)** Scatterplot showing no correlation between egocentric and allocentric scores. **E)** Number of patients showing different levels of accuracy on the Apples Cancellation Test

EN and/or AN were assessed by an expert clinical neuropsychologist (A.M.) using the ACT (Bickerton et al., 2011; Mancuso et al., 2015). This is a highly sensitive test that involves the presentation of 150 upright apples drawn at pseudorandom locations of an A4 sheet of paper (Fig. 1A). One-third of the apples are complete (targets), while the remaining two-thirds are open on either the left or right side (distractors). The midline of the page is positioned along the patient’s midline (Mancuso et al., 2015). Each patient was asked to cross-out all the full apples and to ignore all the open ones, within a maximum time of 5 min.

For each patient, we computed:

1. egocentric score: the number of targets cancelled on the right minus the number of targets cancelled on the left side of the sheet; positive values indicate more targets cancelled on the right than on the left side (left EN), and vice versa for negative values (right EN).
2. allocentric score: the number of left- minus right-open distractors cancelled across the sheet; positive values indicate left AN, and negative values indicate right AN.

Patients were diagnosed with left EN if they showed an egocentric score > 2; instead they were considered to suffer from left AN with an allocentric score > 1 (Mancuso et al., 2015). Consistently with the previous literature (Vallar & Calzolari, 2018), we restricted our investigation to positive scores, excluding from VLSM analysis patients with negative scores (i.e., right neglect; n = 17).

### Neuroimaging assessment

Patients recruited underwent either magnetic resonance imaging (MRI) or computerized tomography (CT), according to the standard stroke protocol of the Santa Lucia Foundation (Table 1; Sperber & Karnath, 2018). Focal lesions were first identified and outlined (using a semi-automated local threshold contouring software; Jim 8.0, Xinapse System, Leicester, UK, http://www.xinapse.com) by an expert radiologist (C.B.), blinded to patients’ condition and performance. For each patient, a binary lesion mask was created (1 for voxels corresponding to a lesion, 0 elsewhere).

Before conducting the VLSM analysis, we normalized the lesion masks to the Montreal Neurological Institute (MNI) space as follows:

1. MR/CT images were firstly skull-stripped using ROBEX (www.nitrc.org/projects/robex; Iglesias et al., 2011);
2. skull-stripped images were then affine transformed with ANTs (Avants et al., 2011) to match the template “MNI152_T1_1mm_brain” (a T1-weighted atlas in MNI coordinates available in the FSL library; www.fmrib.ox.ac.uk/fsl/); the same transformation was then applied to the lesion mask to get it normalized to the MNI space.

**Fig. 2:**
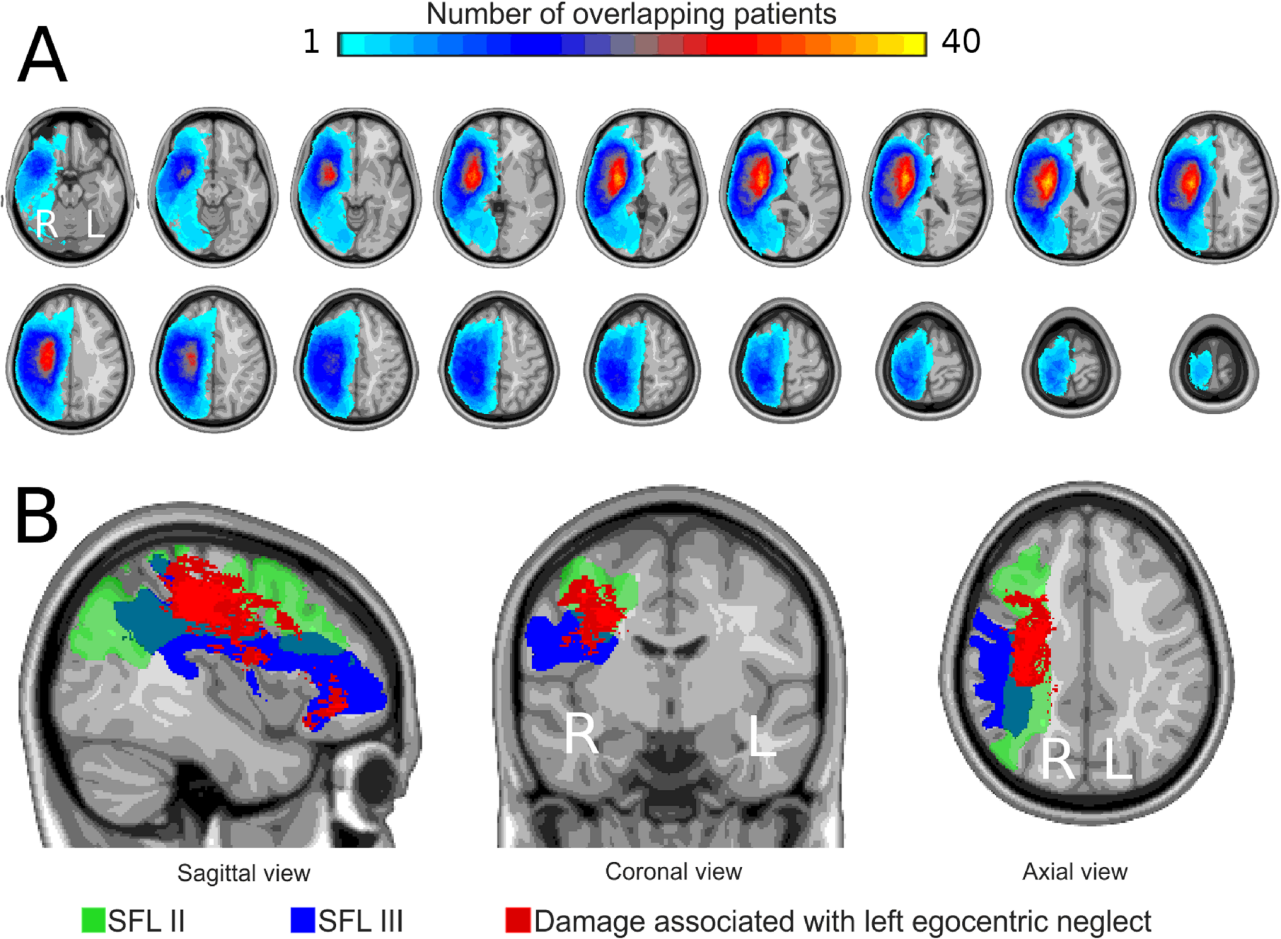
**A)** Lesion maps overlaid onto the axial slices of a T1-weighted atlas, showing lesion up to the maximum overlap (n = 40). Abbreviations: R = right; L = left. **B)** Results of the voxel-lesion symptom-mapping (VLSM) analysis with egocentric scores on the Apples Cancellation Test, overlaid onto a sagittal, coronal and axial sections of a T1-weighted atlas. Regions associated with left egocentric neglect (red map) were located on the anatomical pathways of SLF II (green map) and SLF III (blue map). Abbreviations: SLF II = middle superior longitudinal fasciculus; SLF III = ventral superior longitudinal fasciculus; R = right; L = left

An overall lesion map, indicating the number of patients with a lesion in a given area, was obtained by combining all MNI normalized lesion masks (Fig. 2A).

### VLSM analysis

We used continuous VLSM in a large sample of early subacute right-stroke patients to identify the relationship between left egocentric/allocentric performance on the ACT and the location of brain lesions. VLSM provides an operator-independent measurement of the association between the anatomical localization of brain damage and patients’ performance on a specific cognitive task (Bates et al., 2003). We considered the severity of neglect by including egocentric and allocentric scores as continuous variables. This procedure allowed us to include patients irrespective of the test outcome (pathological/non-pathological). Our VLSM analysis was therefore characterized by a large spectrum of left egocentric/allocentric deficits, ranging from normal to strongly pathological performance. This approach increased the sensitivity of our measures by accounting for the severity of symptoms and not just for their categorical presence (cf. Chechlacz et al., 2010).

Two separate VLSM analyses were performed using the VLSM2 Matlab toolbox (v2.60, https://aphasialab.org/vlsm/) on EN and AN positive scores as variables of interest, using age and sex as covariates (Bates et al., 2003). Two additional and more restrictive analyses were conducted to account for variability in egocentric performance explained by allocentric performance, and vice versa. For this, we included allocentric scores in the egocentric VLSM, and egocentric scores in the allocentric VLSM as an extra covariate. Then, in order to account for the presence of mixed forms of neglect, we conducted another VLSM analysis on patients showing both EN and AN (n = 33), using the sum of both scores as the variable of interest, and age and sex as covariates.

In all these VLSM models, the analysis was restricted to those voxels lesioned in at least 10 out of 76 patients to maintain a reasonable level of statistical power. Voxel-wise significance thresholding was set at p < 0.001, and the resulting t-statistic maps were corrected for multiple comparisons by permutation tests (5000 iterations; Kimberget al., 2007). We accepted as significant clusters set to p-FWE-corrected values < 0.05.

Lesions were anatomically localized using the Duvernoy Human Brain Atlas (Duvernoy, 1991) and the Brain Connections Atlas (Rojkova et al., 2016).

## Results

### Performance on the ACT

Irrespective of their inclusion in the VLSM analysis, 22 out of 100 patients recruited showed non-pathological scores on both egocentric and allocentric ACT, while 78 showed EN and/or AN. 36 showed both left EN and left AN, 1 both right EN and right AN, 4 both left EN and right AN, 6 both right EN and left AN, 13 left EN only, 12 left AN only, 5 right EN only, 1 right AN only (Fig. 1B-C).

Performance on the egocentric and allocentric versions of the ACT were independent, as evidenced by the absence of a correlation between the two scores (Pearson’s *r* coefficient = 0.136, p = 0.176; Fig. 1D). Overall, our sample showed high variability in the accuracy on the ACT (Fig. 1E).

The VLSM analyses included 76 patients with positive scores on both egocentric and allocentric measures. Amongst them, 19 patients (25%) had non-pathological scores, neither on the egocentric nor on the allocentric ACT. By contrast, 57 patients (75%) showed pathological performance on the ACT: 12 (16%) showed only left EN; 12 (16%) showed only left AN, and 33 (43%) showed both left EN and AN.

### VLSM

The VLSM analysis on the left egocentric scores revealed one significant cluster (volume = 34,127 voxels; p-FWE-corrected = 0.0008; Fig. 2B, red map) wherein lesioned voxels were associated with a decreased performance on crossing-out the complete apples on the left half of the sheet (i.e., on the egocentric ACT). Lesion voxels substantially overlapped with the right middle and ventral superior longitudinal fasciculus (SLF II and SLF III, respectively). These results were confirmed by the more restrictive VLSM analysis that included allocentric scores as a covariate. This confirmed the same cluster reported in Fig. 2B, red map (volume = 34,127 voxels; p-FWE-corrected = 0.0014). Conversely, no regions were found to be significantly associated with poor performance on the allocentric version of the ACT, irrespective of the inclusion (or not) of the egocentric scores as a covariate. Finally, we did not find any region significantly associated with a combined egocentric/allocentric performance, as evidenced by the VLSM analysis on patients showing both types of impairment.

## Discussion

This study aimed to unravel the neural substrates associated with left EN and AN by using VLSM analyses in a large sample of consecutive early subacute right-stroke patients. The severity of EN/AN symptoms was simultaneously assessed using the ACT.

We found a significant association between decreased egocentric scores on the ACT and the presence of white matter lesions in the SLF II and SLF III. This result was not affected by patients’ allocentric performance, indicating a selective contribution of these lesions to egocentric performance. Conversely, we did not find any significant association between right-stroke damage and left allocentric or mixed forms of neglect. These findings point to dissociable and independent mechanisms of EN and AN. They agree with a recent behavioural study (Turgut et al., 2017) arguing that EN/AN cannot be explained by a common mechanism, which was evidenced by the absence of any correlation between EN and AN severity (Demeyere & Gillebert, 2019). Consistently, we found no correlation between egocentric and allocentric scores in our cohort of patients, confirming performance independency.

Several anatomical studies support the notion that neglect symptoms are related to white matter injuries, which might contribute to functional disconnection between nodes within attention-related neural networks (Bartolomeo et al., 2007; Nardo et al., 2019; Vaessen et al., 2016). Here, we show a clear association between left EN and white matter lesions within the right SLF (II and III), posteriorly close to the TPJ. These data dovetail with previous evidence pointing to a key role played by the SLF in neglect symptoms (Chechlacz et al., 2012; Molenberghs et al., 2012; Thiebaut de Schotten et al., 2005; Urbanski et al., 2011; Verdon et al., 2010). Our findings extend the previous literature by showing a selective association between SLF damage and EN in a large cohort of early subacute right-stroke patients, over and above any influence of AN.

While we found a clear link between white matter damage and egocentric performance, we failed to detect any brain lesions associated with AN. No associations were observed even when allocentric and egocentric scores were combined. This finding implies that in the current sample there are no specific lesions that are selectively associated to the performance on the allocentric version of the ACT. Conversely, allocentric performance might be more “globally” related to whole brain damage included in the lesion map (Fig. 1A). This finding is in line with a recent lesion mapping study on early subacute right-stroke patients that showed anatomical correlates for peri- and extra-personal EN, but not for AN (Ten Brink et al., 2019). However, these findings are not consistent with Chechlacz et al. (2010), who reported a dissociation between EN and AN, the former associated with more anterior cortical damage and damage within subcortical structures, and the latter associated with damage to more posterior cortical regions. In addition, they reported that damage to the intraparietal sulcus and TPJ was associated with both forms of neglect, as well as with extended white matter damage.

This discrepancy might be explained by several methodological differences. First, Chechlacz et al. (2010) included in their study patients with heterogeneous etiologies (stroke, carbon monoxide poisoning, degenerative changes) and in the chronic stage. Conversely, we included only right-stroke patients at an early subacute stage. Very likely, such differences had a substantial impact on results. Measuring neglect symptoms immediately after stroke (vs. later stages) might result in differences related to immediate spontaneous neurobiological recovery vs. later brain functional reorganization, respectively (Ten Brink et al., 2019). Several authors suggested that neglect might be better conceived as a disconnection syndrome (Bartolomeo et al., 2007; Doricchi & Tomaiuolo, 2003). As such, neglect could be related to white matter damage at an early stage, and to both white and gray matter damage at a chronic phase, when functional reorganization has taken place. This might explain why we found only white matter damage but no evidence of cortical involvement. The fact that we did not find associations with white matter damage in AN in early subacute patients suggest that this form of neglect might be less related to structural brain damages. Future studies might use functional connectivity to better characterize the neural substrate of left AN.

The present study suffers from a potential limitation, namely, the inclusion of both CT and MRI scans. Combining CT/MRI data for lesion analysis studies has become common practice in recent years (Biesbroek et al., 2016; Karnath et al., 2004; Kenzie et al., 2015; Sperber & Karnath, 2018, Ten Brink et al., 2019, Verdon et al., 2010). However, it is possible that the accuracy of lesion delineation might differ between CT and MRI images. Furthermore, the resolution of the CT and MRI scans could affect the precision of the VLSM results (Ten Brink et al., 2019). Nevertheless, we opted for a robust design including as many patients as possible, to optimize statistical power while accepting some heterogeneity in scan acquisition (Ten Brink et al., 2019). In addition, as suggested by de Haan and Karnath (2018), the systematic exclusion of patients with CT images might introduce a selection bias, typically influencing important factors such as lesion size, general clinical status, and severity of cognitive deficits.

## Conclusion

We conducted VLSM analyses on a large sample of early subacute right-stroke patients assessed by the ACT. We found no correlation between egocentric and allocentric performance, pointing to the existence of independent mechanisms supporting the two forms of neglect. This was confirmed by VLSM analyses, showing an association of SLF damage with left EN, but not with AN. These findings, observed in early-stage, subacute patients, highlight the role played by white matter lesions in impaired attention-related operations within an egocentric frame of reference.

## Declarations

This study was approved by the Fondazione Santa Lucia’s Local Ethic Committee (CE/PROG.597) and was conducted in accordance with the Declaration of Helsinki.

All study participants have provided written informed consent.
